# Freeze Dried Quetiapine-Nicotinamide Binary Solid Dispersions: A New Strategy for Improving Physicochemical Properties and Ex Vivo Diffusion

**DOI:** 10.1155/2016/2126056

**Published:** 2016-11-30

**Authors:** Ahmed Mahmoud Abdelhaleem Ali, Mayyas Mohammad Ahmad Al-Remawi

**Affiliations:** ^1^Department of Pharmaceutics and Pharmaceutical Technology, College of Pharmacy, Taif University, Taif, Saudi Arabia; ^2^Department of Pharmaceutics and Industrial Pharmacy, Faculty of Pharmacy, Beni-Suef University, Beni-Suef, Egypt; ^3^Department of Pharmaceutics and Pharmaceutical Technology, Faculty of Pharmacy and Medical Sciences, Petra University, Amman, Jordan

## Abstract

Improving the physicochemical properties and oral bioavailability of quetiapine fumarate (QF) enabling enhanced antipsychotic attributes are the main aims of this research. The freeze dried solid dispersion strategy was adopted using nicotinamide (NIC) as highly soluble coformer. The prepared dispersions were characterized using scanning electron microscopy (SEM) differential scanning calorimetry (DSC), Fourier transform infrared spectroscopy (FTIR), and X-ray diffraction (XRD). Static disc intrinsic dissolution rate and ex vivo diffusion through intestinal tissues were conducted and compared to pure quetiapine fumarate. The results demonstrated a highly soluble coamorphous system formed between quetiapine fumarate and nicotinamide at 1 : 3 molar ratio through H-bonding interactions. The results showed >14-fold increase in solubility of QF from the prepared dispersions. Increased intrinsic dissolution rate (from 0.28 to 0.603 mg cm^−2^ min^−1^) and faster flux rate through duodenum (from 0.027 to 0.041 mg cm^−2^ h^−1^) and jejunum (0.027 to 0.036 mg cm^−2^ h^−1^) were obtained. The prepared coamorphous dispersion proved to be effective in improving the drug solubility and dissolution rate and ex vivo diffusion. Therefore, binary coamorphous dispersions could be a promising solution to modify the physicochemical properties, raise oral bioavailability, and change the biopharmaceutics classification (BCS) of some active pharmaceutical ingredients.

## 1. Introduction

Quetiapine fumarate (QF) is a dibenzothiazepine antipsychotic drug used in treatment of schizophrenia and mania associated with type I bipolar disorders [1]. The drug has a relatively short half-life (6 hr) and undergoes extensive first-pass metabolism. The high frequency of administration of quetiapine (2–4 times daily) often results in numerous side effects including panic attacks, dyspnea, and swelling of lips and face [2]. Quetiapine is marketed under the brand name Seroquel™ in different strengths (25–300 mg) and shown to be effective up to a dose of 750 mg/day [3]. Quetiapine pharmacokinetics may change when concomitantly administered with some drugs such as ketoconazole, phenytoin, or rifampicin which affect the activity of CYP3A4 enzymes [4]. Quetiapine has high affinity to serotonin 5-HT_2_ than dopamine receptors which makes it suitable for successful control of psychotic symptoms associated with Parkinson's disease without worsening of body movements [5]. Quetiapine is soluble in acidic pH (2–4); however, due to its low water solubility over the physiological pH and high permeability, it was classified as BCS class II drug [6]. Therefore, solid state modification approaches which will increase its water solubility are expected also to improve its bioavailability and enable bypassing hepatic metabolism if administered in suitable dosage form such as orodispersible or sublingual tablets or films. The increased solubility by new formulation will enable development of new and predictable quality oral and/or parenteral sustained release dosage forms of the drug.

Stabilized amorphous solid dispersions of poorly soluble active ingredients are considered new emerging technology for improvement of water solubility and bioavailability for biopharmaceutics classification system (BCS) class II drugs. Amorphous polymeric glass solutions were used for long time as the favorable system for improving the solubility, dissolution rate, and stabilization of amorphous drugs [7, 8]. However, these types of systems are hygroscopic and more liable to reduction of glass transition temperature (*T*_*g*_) and recrystallization upon storage [9–11]. Also, large amounts of polymers are often needed to produce effective systems. In addition a predetermined miscibility of the drug with the selected polymer is required [12]. All these challenging problems make it hard to manufacture amorphous dispersions [13–15]. As more promising alternative to polymers, low molecular weight excipients that could interact with the drug and lower the melting point and increase the *T*_*g*_ such as sugars, amino acids, and hydrotropic organic acids are currently used for drug amorphization [9, 16, 17].

Although the above-mentioned alternatives produced highly dissolving amorphous systems, yet some systems showed partial crystallinity observed from remaining unreacted drugs or excipients [18, 19]. Binary amorphous systems that are composed of two pharmacologically related low molecular weight drugs were recently introduced as effective coamorphous dispersions (COADs) that overcome the drawbacks of drug-polymer glass solutions [20, 21]. Many physically stable coamorphous dispersions with high solubility and dissolution rate were reported in the literature such as those formed between nateglinide and metformin, simvastatin and glipizide prepared by ball milling [22, 23], and naproxen and indomethacin by quench cooling [24]. Other methods such as solvent evaporation or spray drying could also be used for COAD formation [25]. However, due to the limited numbers of drug combinations that could interact at the molecular level with hydrogen or ionic bonding and produce coamorphous mixtures, other substitutes to one of the drugs can be made [26].

Amino acids and neutral molecules such as saccharin or nicotinamide, which are often used for cocrystal formation, can also be applied for COAD preparation using fast methods of solvent evaporation [15]. In this research amorphization of QF was undertaken through combination of with nicotinamide in different molar ratios using hot and cold approaches for rapid solvent evaporation in an attempt to enhance oral bioavailability and change the BCS classification of QF. The produced coamorphous dispersions were evaluated by solid state characterization, equilibrium solubility, intrinsic dissolution rate (IDR), and gut permeation compared to the pure drug.

## 2. Materials and Methods

### 2.1. Materials

Quetiapine fumarate (QF) was obtained as free sample from the Jordanian Pharmaceutical Manufacturing, Amman, Jordan. Nicotinamide (NIC) was purchased from Sigma Aldrich, UK. Acetonitrile and methanol (HPLC grades) were purchased from Fluka, UK. Absolute ethanol, sodium dihydrogen phosphate, sodium hydroxide, and phosphoric acid were obtained from Natco Pharma, Hyderabad, India.

### 2.2. Preparation of Quetiapine Fumarate-Nicotinamide Coamorphous Dispersions

**Figure 1:**
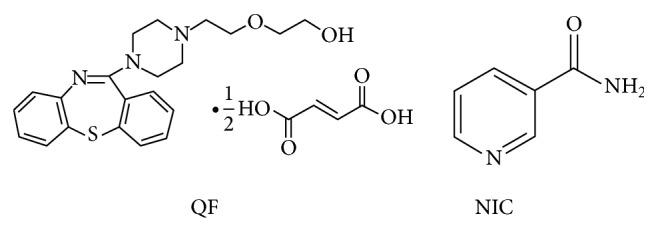
Chemical structure of quetiapine fumarate (QF) and nicotinamide (NIC).

The coamorphous dispersions were prepared by a previously published coprecipitation method using fast solvent evaporation with some modifications [27]. Mixtures of QF and NIC based on their molar mass (441.5 and 122.12 g/mole) and electron donner/acceptor functional groups (Figure 1) were prepared as shown in Table 1. Two solvent systems, ethanol and 50% v/v mixture of ethanol and water (20 mL), were used for dissolving the drug and the conformer using 250 mL round bottom flask. QF dissolves well in ethanol but nicotinamide takes relatively longer time (30 min) on the sonicator before complete dissolution. As the formulations were dried using freeze drying, QF was dissolved in 20 mL ethanol and separately nicotinamide was added to an equal volume of water and the two solutions were admixed just before drying. Freeze drying (cold evaporation) was applied using Christ freeze dryer model Alpha 2–4 LD plus (Osterode, Germany). The fast and controlled cold evaporation increases the chances of contact and molecular H-bonding interaction between quetiapine and nicotinamide in concentrated solutions during freeze drying and could result in amorphous solid dispersions. The obtained powders were kept under completely dry conditions using silica gel desiccators until further investigation.

### 2.3. Scanning Electron Microscopy (SEM)

The morphology of the prepared dispersions was examined using scanning electron microscopy (Analytical Scanning Microscope, JEOL-JSM-6510LA, JEOL, Japan). Few specks from each formulation were placed on the carbon stubs and then coated using a gold sputter (SPI-Module Sputter Coater, SPI Supplies Inc., USA) followed by microscopical scanning.

### 2.4. Differential Scanning Calorimetry (DSC)

Samples of pure QF and NIC and the prepared COAD formulations (5 mg each) were individually filled into aluminum flat bottomed pans and heated using a simultaneous thermogravimetry-differential scanning calorimeter model STA 449 F3 Jupiter (Nietzsche, Germany) in an atmosphere of nitrogen. The heating temperature used for the crystalline components was set between 20 and 300°C with a heating rate 10°C min^−1^. The percentage crystallinity *Xc* (%) of the prepared samples was calculated from the enthalpy of fusion of the sample (Δ*H*) compared to that of 100% crystalline QT (Δ*H*_0_) according to the following equation [28]:

(1) $$\begin{array}{c} Xc\left(\;\%\;\right)=\left(\;\frac{\Delta H}{\Delta H_{0}}\;\right)\times 100. \end{array}$$

### 2.5. Fourier Transform Infrared Analysis (FTIR)

Small samples (2-3 mg) of QF and NIC and the prepared COADs were individually mixed with 500 mg dry potassium bromide. The powder mixtures were compressed into discs under a pressure of 68.5–103.4 MPa using a hydrostatic press. The infrared spectrum was determined at a scanning range of 1000–3500 cm^−1^ to detect major characteristic bands in QT and NIC using a Fourier transform infrared instrument (IR Prestige-21, Shimadzu, Japan).

### 2.6. X-Ray Diffraction (XRD)

Samples of QF powder and NIC as well as the COAD formulations were subjected to X-ray diffraction analysis. A Shimadzu XRD-6000 X-ray powder diffractometer (Shimadzu, Japan) coupled with a standard Cu sealed X-ray tube with voltage, current (40 kV and 40 mA), was used to characterize the amorphous or crystalline state of formulations [29]. Data collection was performed at 2*θ* of 5–60° in steps of 0.04 and scanning speed of 0.4 degrees per step. Any change in the crystalline pattern of the prepared coamorphous dispersions compared to those of the parent crystalline components was recorded and evaluated. The index of crystallinity was calculated according to the ratio of the relative intensity of the sample (*Is*) compared to that of the pure crystalline component (*Ic*) with highest peak [30, 31] according to the following equation:

(2) $$\begin{array}{c} \%\text{ Crystallinity index}=\left(\;\frac{Is}{Ic}\;\right)\times 100. \end{array}$$

### 2.7. Drug Content and Equilibrium Solubility

Samples of the prepared amorphous dispersions equivalent to 5 mg QF were dissolved in methanol and adjusted to volume using a standard 50 mL volumetric flask. Then, 2 mL was taken and diluted to 10 mL with mobile phase. Drug content was then determined using a photodiode array automated HPLC analysis system model DGU-20A3/LC-20AT/SIL-20A/CTO-20A/SPD-M20A (Shimadzu, Japan). For equilibrium solubility, an amount of the prepared COAD formulations equivalent to 10 mg QF was weighed, placed into 2 mL Eppendorf tubes, and dispersed into 250 *μ*L of distilled water. The dispersions were then placed on an orbital shaker model SSM (Stuart, UK) operated at a rate of 300 cycles per min. The process was continued for 72 hr followed by sonication for 30 minutes; then, 10 *μ*L of the filtered supernatant was taken and diluted to 10 mL with methanol. The diluted sample solutions (*n* = 3) were measured by HPLC analysis. The mobile phase was composed of a mixture of acetonitrile and 0.02 M phosphate buffer (50 : 50 v/v) at pH 5.5 adjusted by 0.02 M orthophosphoric acid and 0.02 M NaOH. The flow rate was adjusted to 0.8 mL/min and the detector wave length was set at 247 nm [32].

### 2.8. In Vitro Intrinsic Dissolution Rate Studies

Fixed-disc method was applied to determine the intrinsic dissolution rate [33]. In this test, an amount of pure QF (15–20 mg) and an equivalent amount from the selected COAD formulations (F6) were compressed into small tablets inside aluminum discs (0.4 cm in diameter) using a manual mini hand press model MHP-1 (Shimadzu, Japan). The backs of the discs (*n* = 3) were covered with a layer of molted hard paraffin which were permitted to solidify before immersion into the dissolution medium. The study was performed using full automated dissolution system model UDT-804 paddle dissolution apparatus (Logan, USA) with vessels containing 500 mL of phosphate buffer pH 6.8 and rotation speeds of 50, 75, and 100 rpm in three runs. The temperature of the media was kept at 37 ± 0.5°C and at predetermined time intervals (15, 30, 60, 90, and 120 min); samples were automatically withdrawn and taken for HPLC analysis. The intrinsic dissolution per unit area of the disc (*G*_*ω*_) was determined as a function of dissolution time and the intrinsic dissolution rate at infinite rotation speed (*K*_1_), that is, at zero diffusion layer (mg·cm^−2^·sec^−1^), was obtained from the following equation resulting from plotting the reciprocal of *G*_*ω*_ against the reciprocal of the angular velocity *ω* [34]:

(3) $$\begin{array}{c} \frac{1}{G_{\omega }}=\frac{1}{K_{1}}+K_{2}\frac{1}{\omega }, \end{array}$$

where *G*_*ω*_ is the intrinsic dissolution rate at *ω* (mg·cm^−2^·sec^−1^), *K*_1_ is the intrinsic dissolution rate at infinite rotation speed (mg·cm^−2^·Sec^−1^), *K*_2_ is a constant, and *ω* is the angular velocity of the disc (radians·sec^−1^).

### 2.9. Ex Vivo Diffusion Studies

The test was performed to compare the rate of diffusion of pure QF to that of the prepared coamorphous dispersions using parts of a fresh cattle gut. The excised tissues of duodenum and jejunum were placed on 3.5 cm in diameter plastic diffusion cells (SES GmbH, Germany) filled to top with 8 mL phosphate buffer (pH 6.8) containing 20 mg pure drug or equivalent amount of the dispersion. The units were placed at the bottom of the dissolution flasks containing 250 mL of the buffer solution. The paddles were rotated at a speed of 100 rpm and the temperature was adjusted to 37 ± 0.5°C. After 0.5, 1, 2, 3, 4, 5, and 6 hr intervals samples were automatically withdrawn and taken for HPLC analysis using the same method mentioned under the drug content and equilibrium solubility section. The flux rate *J*_0_ (mg·cm^−2^ hr^−1^) was obtained from the slope of the plotted line relating cumulative amount of QF permeated (mg/cm^2^) to time (hr) as shown in Figure 8 [35].

## 3. Results

### 3.1. Solid State Characterization 

#### 3.1.1. DSC Studies

**Figure 2:**
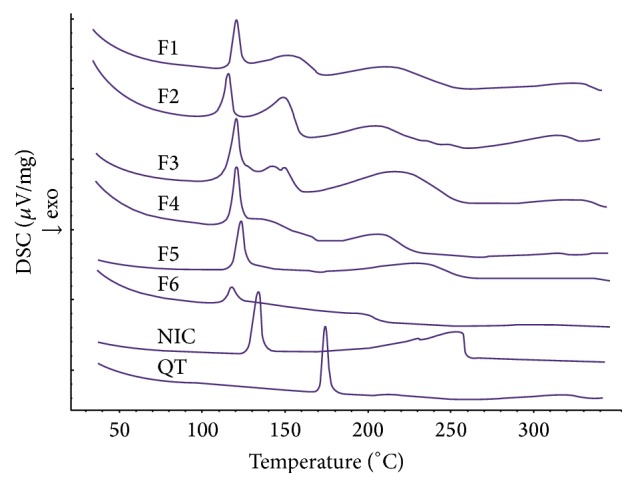
DSC thermograms of quetiapine fumarate (QT), nicotinamide (NIC), and solid dispersions (F1–F6).

The DSC thermograms of QF and NIC and the solid dispersions are shown in Figure 2. Characteristic melting peaks at specific temperatures for QF and NC were observed at 175.40 and 131.80°, respectively. The prepared dispersions showed lower melting temperatures compared to parent components as displayed in Table 2 containing the thermal data of QF/NIC coamorphous dispersions. From Table 2, it could be noticed that two peaks with different melting points were recorded with various heats of fusion (Δ*H*_*f*_) from the DSC thermograms of COAD formulations F1, F2, and F3 whilst single peaks were observed for F4, F5, and F6. In addition, the sharp decrease in weight was monitored using TGA to indicate the decomposition temperature. The coamorphous dispersion F6 showed the lowest melting temperature (117°C), lowest heat of fusion (Δ*H*_*f*_ = 31 *μ*V/mg), and decomposition temperature (172°C). The % crystallinity was also calculated from the DSC data and the results showed that the dispersion F6 (1 : 3  Mr) demonstrated the lowest crystallinity (19%) as shown in Table 2.

#### 3.1.2. FTIR Studies

**Figure 3:**
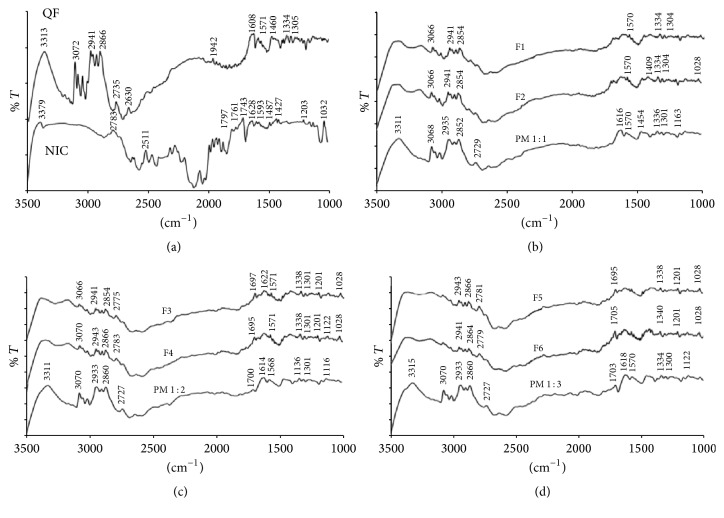
IR spectra of quetiapine fumarate (QF), nicotinamide (NIC), physical mixtures (PM1–3), and the prepared dispersions (F1–F6).

FTIR results collected for COAD samples as well as pure QF and NIC were shown in Figure 3. The FTIR spectra of NIC indicated the appearance of the characteristic amino group –NH2 symmetric stretching vibrations at 3379 cm^−1^ (Figure 3(a)). The C=O stretching also appeared at 1628 and the –CN stretching vibrations also appeared at 1427 cm^−1^ which correspond well with the recorded values in the literature [36]. Other characteristic peaks were noticed such as the amide band at 1703 and C–O stretching at 1032 cm^−1^. The FTIR spectra of QF (Figure 3(a)) showed characteristic peaks at 3313, 3072, 3045, 3012, 2941, 2866, 2870, 2738, 2627, 2347, 1942, 1608, 1568, 1460, 1342, and 1305 cm^−1^. These peaks also are highly similar to those of QF Form I polymorph reported in the literature [37]. The peak at 3313 corresponded to –OH stretching vibrations, between 3072 and 3130 cm^−1^ corresponding to –CH aromatic stretching, whilst –CH aliphatic stretching vibrations were observed at bands in the region between 2900 and 2940 cm^−1^. The aromatic amino (secondary and tertiary) showed absorption bands between 1300 and 1340 cm^−1^. The fumarate salt moiety showed characteristic broad –OH stretching band which overlapped the –CH stretching region between 300 and 3300 cm^−1^. The prepared dispersions showed IR bands shorter than those of parent components and also demonstrated shifting of some peaks and formation of shoulders in others as observed in Figures 3(b)–3(d).

#### 3.1.3. XRD Studies

**Figure 4:**
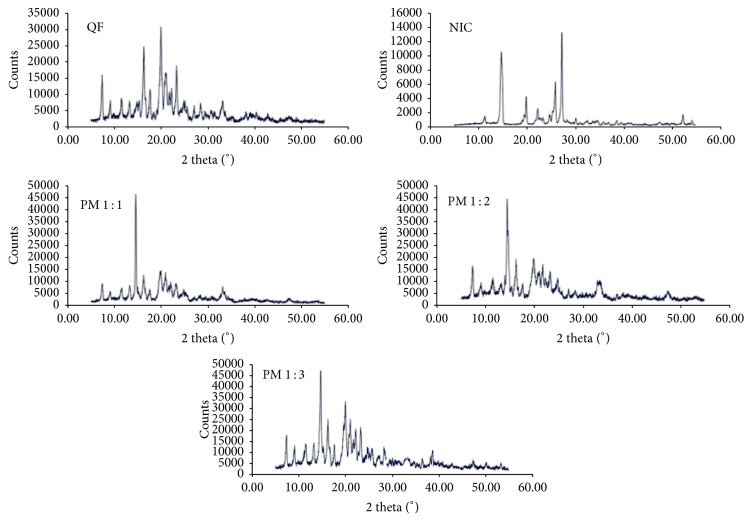
X-ray diffractograms of quetiapine fumarate (QF), nicotinamide (NIC), and physical mixtures (PM1–3).

**Figure 5:**
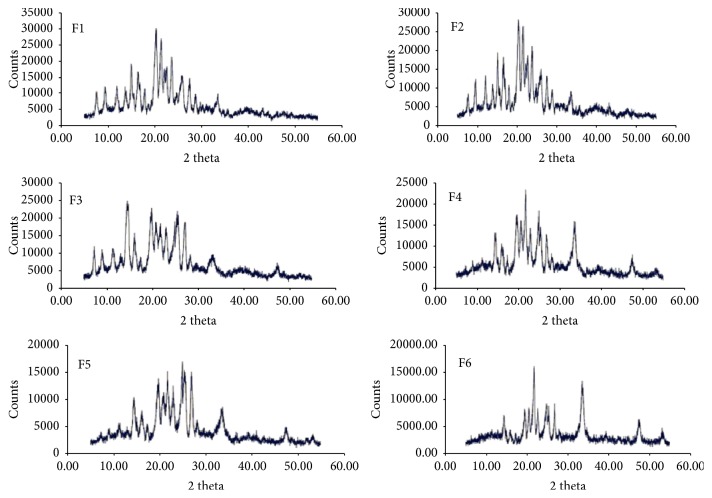
X-ray diffractograms of QF-NIC binary coamorphous dispersions (F1–F6).

The results of XRD data collected for QF and NIC and the coamorphous samples are shown in Figures 4 and 5. The highly crystalline QF showed sharp diffraction lines at 2*θ* 7.3, 9.1, 11.5, 13.2, 14.9, 15.3, 16.2, 17.6, 19.9, 21, 21.7, 22.3, 23.2, 24.8, 25.1, 25.5, 27.1, 28.4, 29.2, 30.6, 33.1, 40.3, and 42.7 degrees. The XRD pattern of NIC showed characteristic diffraction lines at 2*θ* values of 21.67°, 22.63°, 24.81°, 26.43°, 29.91°, 31.57°, 33.55°, 35.88°, 37.52°, and 40.28° (Figure 4). The degree of crystallinity was obtained by calculating the ratio of highest intensity of the solid dispersion sample to that of the pure crystalline component (Table 2). The XRD patterns of the dispersions shown in Figure 5 demonstrated lower intensity (counts 25000–15000) compared to the physical mixtures (counts >45000). It was observed that sample F6 showed the lowest degree of crystallinity (24.0%) compared to other dispersions (Table 2).

### 3.2. Morphology of the Prepared Dispersions Compared to Parent Components

**Figure 6:**
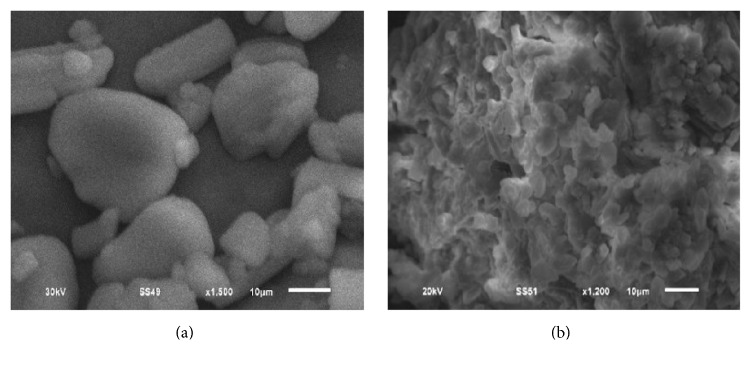
Scanning electron micrographs (SEM) of QF-NIC physical mixture (a) and binary coamorphous dispersion F6 (b).

Scanning electron micrographs of QF and NIC (Figure 6) demonstrated crystalline shape of both compounds. The SEM images of the physical mixture showed small rod like crystals of QF dispersed on large prismatic crystals of NIC (Figure 6(a)). The binary coamorphous dispersion F6 (prepared at 1 : 3 molar ratio) showed lack of defined crystals and appearance of a homogenous dispersion lacking defined crystals (Figure 6(b)).

### 3.3. Drug Content and Equilibrium Solubility

**Figure 7:**
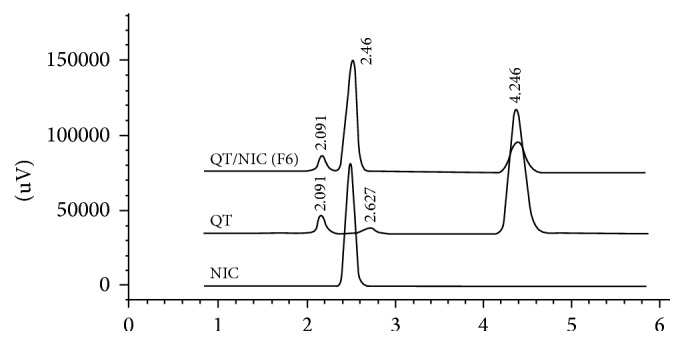
HPLC chromatograms of QF and NIC and the binary coamorphous dispersion (F6) after dissolution.

The results of measured equilibrium solubility of the prepared dispersions obtained after HPLC analysis are shown in Table 3. The analysis data showed separate and sharp peaks for both components after injection of the dispersion formulation (Figure 7) indicating chemical stability of separated QF from the dispersion. Formula F6 composed of QF and NIC in 1 : 3 molar ratio and prepared by cold evaporation method demonstrated the highest relative percentage increase in QF water solubility (1363%) with more than 16-fold increase in concentration compared to the pure crystalline QF (Table 3). Formula F5 which had similar composition and evaporation method to F6 also showed comparable percentage increase in solubility (1303%). The drug content per 5 mg of the dispersion was calculated for each formula and the results were found to be comparable to the theoretical content with insignificant differences between the two (*P* > 0.05).

### 3.4. Intrinsic Dissolution Rate and Ex Vivo Diffusion Studies

**Figure 8:**
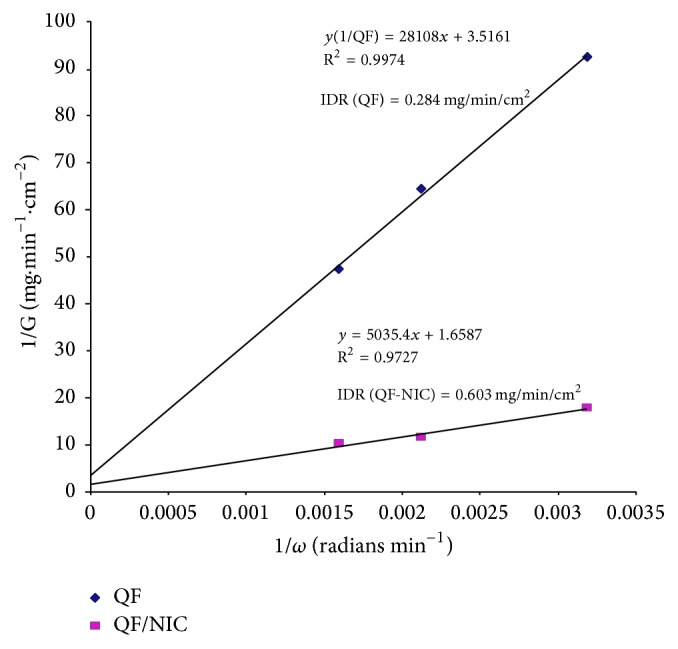
Plot of the reciprocal of the dissolution rate 1/*G*  (mg cm^−2^ min^−1^) versus reciprocal of angular velocity (1/*ω*) (radians·min^−1^) for determination of IDR from QF and coamorphous dispersion F6 (QF-NIC).

The calculated IDR for pure QF and F6 under the above-mentioned experimental conditions was found to be 0.284 and 0.603 mg·cm^−2^·min^−1^, respectively, indicating more than twofold increase in IDR (Figure 8).

**Figure 9:**
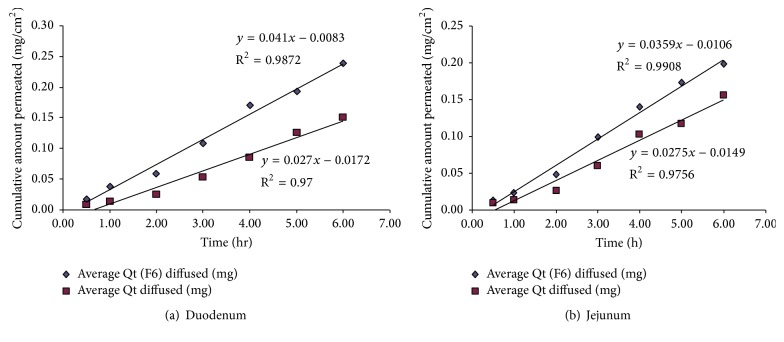
Ex vivo gut permeation profile of quetiapine fumarate (QF) compared to the binary coamorphous dispersion (F6).

The in vitro diffusion studies through cattle intestinal sections (duodenum and jejunum) showed superior and faster diffusion of QF from the dispersion F6 compared to pure QF (Figures 9(a) and 9(b)). Significantly different (*P* < 0.05) flux rates (*J*_0_) for the new dispersion (0.041 mg cm^−1 ^h^−1^) compared to pure QF (0.027 mg cm^−1 ^h^−1^) through duodenum and (0.036 mg cm^−1^ h^−1^ to 0.028 mg cm^−1^ h^−1^) for jejunum were observed, respectively (Table 4). The data also showed statistically significant difference in the lag times prior to commencement of diffusion in both cases in favor of the F6 which showed substantially lower lag times than pure QF and the reported values in the literature [38].

## 4. Discussion

The crystalline nature of QF and NIC was confirmed by sharp melting endotherms which were found to coincide with those previously published 174°C [39] and 128–131°C [40], respectively. The COAD F6 demonstrated a single short endotherm with minimum melting temperature indicating miscibility of the two components [41]. The lowest heat of fusion (31 *μ*V/mg) of this formula among other prepared COADs may also indicate that such preparation could have the maximum degree of amorphousness. The IR spectra showed that the physical mixtures had much shorter peaks which represent summation of the peaks of QF and NC with possible interactions (Figure 3). The binary dispersion samples, however, showed much broader and shorter peaks and disappearance of some characteristic peaks of QF. The characteristic peaks at 3313 of QT and 3379 cm^−1^ of NIC which represent –OH and –NH stretching vibrations were found to form a bridge in all binary solid dispersions (Figures 3(b)–3(d)) indicating H-bonding interactions [42]. Complete disappearance of the characteristic amide peaks of NIC between 1628 and 1797 cm^−1^ and C=O asymmetric stretching of QT salt at 1608 cm^−1^ have been observed in almost all dispersions also suggesting H-bonding formation [43, 44]. The coamorphous sample F6 (Figure 3(d)) showed shifting of the characteristic peaks of QT from 2735 to 2775 which may support the suggestion of formation of a true coamorphous dispersion. However, in order to get another proof of amorphousness, the degree of crystallinity was calculated from both DSC and X-ray diffraction data. From the XRD diffraction pattern of QF (Figure 4), it was shown that the diffraction lines were highly identical to those observed for QF Form I polymorph cited in the literature [45]. For nicotinamide also, numerous diffraction lines were similar to those found in previous research works [46]. For the prepared dispersions (Figure 5) the intensity of the diffraction lines was highly shortened especially with the dispersions formulated at 1 : 2 and 1 : 3 molar ratios (F5 and F6). The lowest degrees of crystallinity (31%) observed for F6 from XRD data and 19% as obtained from DSC data support the point of view of formation of substantially amorphous or semicrystalline dispersion [47]. Also the obtained data from SEM (Figure 6) indicated differences in the shape of the final coamorphous dispersion being a homogenous aggregate with undefined edges compared to the clearly identified crystalline parent components. This dispersion also showed the highest equilibrium solubility with more than 14-fold increase in solubility compared to pure crystalline QF.

Therefore, by combining the results of DSC, FTIR, equilibrium solubility, and XRD data, it becomes clear that the coamorphous dispersion F6 had a single and short DSC endotherm, lowest melting temperature, lowest heat of fusion, shorter and broader IR bands, and lowest degree of crystallinity. Hence, this QF/NIC (1 : 3 Mr) coamorphous dispersion could be considered the best obtained amorphous sample with limited crystallinity and consequently it was selected for in vitro intrinsic dissolution and ex vivo diffusion studies.

For equilibrium solubility, F6 obtained the largest value followed by F5 which also support the above hypothesis of COAD formation. The difference between F6 and F5 is in the solvent used during preparation of the dispersion, being ethanol and ethanol/water for F5 and F6, respectively. This small difference in solubility (both showed >14-fold increase in solubility) may indicate that the ethanol/water solvent system has higher support to formation of H-bonding during freeze drying leading to formation of a highly soluble dispersion. The differences in the degrees of crystallinity of F5 (38.6%) compared to F6 (31%) also confirm that the molar ratio 1 : 3 was the best between other ratios (Tables 1 and 2).

The importance of the calculation of the intrinsic dissolution rate (IDR) of drugs evolves from the roles of both drug solubility and IDR in the FDA updated determination of biopharmaceutics classification system of drugs [48, 49]. Although the results of IDR obtained for QF and COAD F6 were found to be a bit lower than the universally suggested values of IDR (1.00 mg·cm^−2^·min^−1^ or 0.017 mg·cm^−2^·Sec^−1^) for drugs classified as highly soluble [38, 50], yet the COAD system showed promising results by approaching that value (Figure 8 and Table 4). It is well known that the measurement of IDR of drugs may vary by variation of the dissolution conditions such as the media composition, pH, and volume; however under the same testing conditions differences between F6 and the reference pure QF were highly evident. In a similar study but under somewhat different conditions, quetiapine benzoate IDR was tested in comparison with quetiapine hemifumarate (using 900 mL degassed water and US Pharmacopoeia 2 apparatus with device for intrinsic testing, at 100 rpm). In this test, quetiapine benzoate demonstrated more than 6-fold increase in IDR compared to quetiapine hemifumarate [51]. In another study for determination of IDR, numerous active pharmaceutical ingredients in the salt form demonstrated IDR values which were not substantially higher than that observed for the prepared COAD [52]. In this study, nicotinamide has been selected as a coformer due to its high solubility, neutral structure, low melting temperature, and capability of interacting at molecular level with H-bonding formation. Such properties were used to obtain stable coamorphous dispersions. The benefits of increased solubility and dissolution rate were tested for impact on the drug diffusion through gut tissues. The results of ex vivo diffusion through duodenum and jejunum showed faster rate of diffusion from F6 (>51%) and >28% compared to QF, respectively (Figure 9 and Table 4). Also, a lower lag time was observed indicating that the new combination is expected to have successful role in improving the oral bioavailability following in vivo administration.

## 5. Conclusions

The above results of preparation and characterization of QF/NIC coamorphous dispersions indicated feasibility of the preparation method and applicability of the dispersion at a molar ratio of 1 : 3 in improving the physicochemical properties of QF. The increased solubility by 14-fold and intrinsic dissolution rate of the drug by 22-fold can be considered an important achievement that will inevitably result in enhanced oral bioavailability. This new formulation could also be used as in-mouth instantly dissolving dosage form which could enable bypassing of first-pass metabolism. The improved ex vivo diffusion (high flux and low lag time) observed with the COAD also provides another proof of success for this new formulation in raising the expected bioavailability and possibly changing of the FDA BCS classification of QF.

## Figures and Tables

**Table 1:** Molar ratio composition of QF-NIC coamorphous dispersions.

Formula number	Drug (mg)	Nicotinamide (mg)	^*∗∗*^Mr	Solvent system	Evaporation method
F1	150	41.49	1 : 1	Ethanol	Cold evap.
F2	150	41.49	1 : 1	^*∗*^Eth/W	Cold evap.
F3	150	82.98	1 : 2	Ethanol	Cold evap.
F4	150	82.98	1 : 2	^*∗*^Eth/W	Cold evap.
F5	150	124.47	1 : 3	Ethanol	Cold evap.
F6	150	124.47	1 : 3	^*∗*^Eth/W	Cold evap.

^*∗*^: Eth/W: ethanol/water system 50 : 50 v/v; ^*∗∗*^Mr: molar ratio.

**Table 2:** Thermal analysis and % crystallinity data of QF coamorphous dispersions compared to pure QF and NIC.

Formula	DSC				TGA	^*∗∗*^Degree of crystallinity (%)	
	Peak 1		Peak 2				
	Melting maximum (°C)	Heat of fusion (*μ*V/mg)	Melting maximum (°C)	Heat of fusion (*μ*V/mg)	Onset of degradation (°C)	DSC	XRD
F1	120.10	37.77	152.00	60.14	186.00	67.34	51.00
F2	117.0	31.42	150.60	65.89	184.00	66.93	53.40
F3	120.00	38.70	142.30	21.67	192.60	39.63	73.30
F4	119.50	73.20	^*∗*^NA	^*∗*^NA	182.70	48.05	51.50
F5	122.00	128.40	^*∗*^NA	^*∗*^NA	199.50	81.69	38.60
F6	117.0	31.0	^*∗*^NA	^*∗*^NA	172.00	19.72	24.00
QF	175.40	134.60	^*∗*^NA	^*∗*^NA	267.30	—	—
NIC	131.80	184.40	^*∗*^NA	^*∗*^NA	200.00	—	—

^*∗*^NA: not available; ^*∗∗*^degree of crystallinity (%) calculated from DSC and XRD data.

**Table 3:** Results of equilibrium solubility of QF coamorphous dispersions compared to pure QF.

Formula	HPLC peak area	Average concentration (mcg/mL ± SD)	Number of folds' increase in solubility	% Increase in solubility (% ± SD)	Theoretical drug content/5 mg dispersion	Actual drug content/5 mg dispersion
F1	569475	10.22 ± 0.102	5.97	497 ± 2.15	3.92	3.94 ± 0.001
F2	578628	10.38 ± 0.337	6.07	507 ± 7.07	3.92	3.89 ± 0.002
F3	963690	17.25 ± 0.080	10.09	909 ± 1.67	3.22	3.18 ± 0.002
F4	792933	14.20 ± 0.076	8.31	731 ± 1.60	3.22	3.21 ± 0.001
F5	1341763	23.99 ± 0.000	14.03	1303 ± 0.00	2.73	2.71 ± 0.003
F6	1398833	25.01 ± 0.032	**14.63**	1363 ± 0.66	2.73	2.74 ± 0.002
^*∗*^QF	92868	1.71 ± 0.035	1.00	0.00	5.00	4.98 ± 0.001

^*∗*^Quetiapine  fumarate.

**Table 4:** Results of in vitro gut permeation of QF through duodenum and jejunum compared to the coamorphous dispersion F6.

Formula	Duodenum			Jejunum		
	Flux rate (mg/cm^2^/h)	Lag time (min)	Regression *R* ^2^	Flux rate (mg/cm^2^/h)	Lag time (min)	Regression *R* ^2^
Pure QF	0.027	38.22	0.97	0.028	32.51	0.98
QF-NIC (F6)	0.041	12.15	0.99	0.036	17.72	0.99
